# Genetic variations of *IL10* and *IL6R* genes in acute anterior uveitis in Han Chinese

**DOI:** 10.1186/s12886-024-03495-6

**Published:** 2024-05-31

**Authors:** Lin Li, Fuzhen Li, Jiankang Shan, Kunpeng Xie, Pengyi Zhou, Haiyan Zhu, Xuemin Jin, Liping Du, Peizeng Yang

**Affiliations:** 1 Department of Ophthalmology, Henan International Joint Research Laboratory for Ocular Immunology and Retinal Injury Repair, The First Affiliated Hospital of Zhengzhou University, Henan Province Eye Hospital, Zhengzhou, Henan P.R. China; 2 The Academy of Medical Sciences, Zhengzhou University, Zhengzhou, P.R. China

**Keywords:** Acute anterior uveitis, *IL6* receptor, *IL10*, Genetic variation

## Abstract

**Background:**

Several autoimmune disorders have been linked to polymorphisms in *IL10* and *IL6R* genes. This research aimed to study whether single nucleotide polymorphisms (SNPs) in the genes of *IL10* and *IL6R* were associated with acute anterior uveitis (AAU) in Han Chinese.

**Methods:**

Genotyping was carried out by the iPLEX Gold Genotyping Assay. Our study comprised 420 patients with AAU and 918 healthy subjects from Han Chinese. Using the chi-square (χ2) test, alleles and genotypes were analyzed between AAU subjects and healthy controls.

**Results:**

All ten SNPs were successfully genotyped and four SNPs (*IL10*/rs1800871, *IL10*/rs3021094, *IL10*/rs2222202, *IL6R*/rs4845618) exhibited weak associations with AAU, as indicated by their P_uncorr_ values. However, upon applying the Bonferroni correction, there was no significant association between AAU and the control subjects. Additionally, the haplotype analysis of the ten SNPs revealed no association with AAU.

**Conclusion:**

Our findings suggested that polymorphisms of the tested ten SNPs on the *IL10* and *IL6R* genes did not show any association with the risk of developing AAU among the Han Chinese population.

## Background

Uveitis may result in significant visual impairment. The incidence of this condition differs globally, with a rate of 200 per 100,000 in North America and 111.3 per 100,000 in Taiwan [1, 2]. Acute anterior uveitis (AAU) is a common form of uveitis [3, 4]. It is characterized by sudden eye pain, redness, and photophobia, and is more prevalent in young to middle-aged adults, with a slightly higher incidence in females than males [3, 5]. While the etiology and pathogenesis of AAU is still unclear, previous studies suggested the involvement of immune responses and genetic factors. Recent studies identified the associations of *HLA-B27*, *FOXO1*, *TRAF5* and *TNFSF15* with AAU [6–8]. However, the exact genetic susceptibility to the disease is not entirely clear.

Genetic variations in interleukin-10 (*IL10*) and interleukin-6 receptor protein (*IL6R*) genes are identified to be associated with autoimmune disorders. Interleukin-10 (IL10) act as an anti-inflammatory cytokine that modulates immune response in autoimmune diseases including psoriasis and uveitis [9, 10]. Several investigations have discovered that *IL10* gene polymorphisms played a role in a wide range of autoimmune disorders including inflammatory bowel disease (IBD), Behcet’s disease (BD), systemic sclerosis (SS) and ankylosing Spondylitis (AS) [11–14]. IL6R is commonly expressed on the cell surface and exists in a soluble form as well. Dysregulation of IL-6/IL6R pathway can increase immune cell activation and the secretion of pro-inflammatory cytokines, resulting in persistent inflammation and damage to tissues [15, 16]. Multiple studies have linked variations in the *IL6R* gene to several conditions, including multiple sclerosis (MS), rheumatoid arthritis (RA), type I diabetes (T1D), IBD and AS in multiple studies [17–20]. Furthermore, a GWAS study in a Caucasian population indicated that *IL10* and *IL6R* were associated with the onset of AAU in AS and IBD, reaching a suggestive level of significance [21].

In light of the aforementioned findings, we undertook a study to explore whether SNPs covering the *IL10* and *IL6R* genes influence the susceptibility to AAU in Han Chinese.

## Methods

### Patients and controls

To identify disease susceptibility loci in the *IL10* and *IL6R* genes, 420 AAU subjects and 918 healthy participants were enrolled in the present research. AAU and healthy subjects were all Han Chinese. All the blood samples were from the patients and healthy controls referred to the first affiliated hospital of Chongqing medical university and the first affiliated hospital of Zhengzhou university from November 2016 to March 2021. AAU subjects were all in acute episodes and had no other autoimmune conditions. Patients with AAU were diagnosed on the basis of symptoms such as blurred vision, red eyes, and ocular pain, as well as clinical examinations that showed ciliary congestion, anterior chamber cells, and keratic precipitates. Our study features a control group of 918 individuals, none of whom have AS, AAU, or any additional autoimmune diseases. our study adhered to the Helsinki Declaration and received approval from the Ethics Research Committees of aforementioned universities. Additionally, all participants provided written informed consent to take part in the study.

### SNP selection

According to prior research, ten tag SNPs were considered for the association study: *IL10*/(rs1800871, rs3790622, rs1800896, rs3021094 and rs2222202) and *IL6R*/( rs2228145, rs6690230, rs4845618, rs4845374 and rs4129267) and alleles and genotype data of the SNPs were all present in single nucleotide polymorphisms database (dbSNP) of the National Center of Biotechnology Information (NCBI) in Han Chinese. Each of the ten loci had a minor allele frequency (MAF) greater than 0.05. Genotyping data for *IL6R* and *IL10* genes in Han Chinese was obtained from HapMap Website (http://hapmap.ncbi.nlm.nih.gov/Index.html.en).

### DNA extraction and genotyping test

The QIAmp DNA Blood Mini Kit (Qiagen Inc., CA, USA) was utilized to extract genomic DNA from peripheral whole blood. Following extraction, DNA samples were kept at -80^°^C. *IL10*/(rs1800871, rs3790622, rs1800896, rs3021094 and rs2222202) and *IL6R*/( rs2228145, rs6690230, rs4845618, rs4845374 and rs4129267) were genotyped by iPlex Gold Genotyping Assay. The MassARRAY Assay design program was utilized to design the primers listed in Table 1.

**Table 1:** Primers used for genotype analysis of *IL10* and *IL6R* genes

Gene	SNP	1st-PCR Primers	2nd-PCR Primers	UEP_SEQ
	rs1800871	ACGTTGGATGATGCTAGTCAGGTAGTGCTC	ACGTTGGATGGTACAGTAGGGTGAGGAAAC	GACCTTGTACAGGTGATGTAA
	rs3790622	ACGTTGGATGTTCTCCCCACTGTAGACATC	ACGTTGGATGTGCTTAGAGCGTTTCCAGAC	CATTAGAAGGAGCTTCTTAAT
***IL10***	rs3021094	ACGTTGGATGGGATTCAACAGTGATGGGAC	ACGTTGGATGTGTAGCTCCGCAGAAAGAAG	CACTTAAATCAGGTCCTCC
	rs2222202	ACGTTGGATGAAGGCATGGGGAGCATCTTC	ACGTTGGATGGGTGAGTTAAGCTAAGCCAG	GGCTAGGAGAAGTAAAGAAA
	rs1800896	ACGTTGGATGTCTGTGGCTGGAGTCTAAAG	ACGTTGGATGGACAACACTACTAAGGCTTC	CTCCCTATCCCTACTTCCCC
***IL6R***	rs6690230	ACGTTGGATGCAGGTTGGCTGGTTTCAAAG	ACGTTGGATGTGGAAGCAATCTCTGTCCTC	TTCAAAAGGAAAGCTCAC
	rs2228145	ACGTTGGATGTTTGAGGCTTTTGACAGCAC	ACGTTGGATGATGTGGGCAGTGGTACTGAA	TGTTGGCAGTGGTACTGAAGAAGAA
	rs4845374	ACGTTGGATGTTTGAGGCTTTTGACAGCAC	ACGTTGGATGATGTGGGCAGTGGTACTGAA	CTTCCTCCTCTATCTTCAA
	rs4845618	ACGTTGGATGGAAAACCTTAGATGACCGGC	ACGTTGGATGCAGATACCCTTCACACTTCC	ACTCCATGATGGCCTTATATCT
	rs4129267	ACGTTGGATGATGGAGAAATACTGGGAGGG	ACGTTGGATGTTCTCTACCTTTGCTGCACC	GAGTGGGGTCAATTCT

### Statistical analysis

Direct enumeration was applied to calculate the allelic and genotypic frequencies. A Chi-Square (X^2^) test was utilized to determine HWE. SNP variants were evaluated with the Fisher Precision or SPSS 2 test in SPSS (version 19.0) to identify allele and genotypic differences. Estimation of the linkage disequilibrium (LD) was performed by calculating R2 and D′ values, while haplotype construction was carried out through the utilization of SHEsis (Shi and He, 2005). The Bonferroni correction method was utilized to alter P values, and a corrected P-value (Pc) of less than 0.05 (Pc<0.05) was considered as statistically significant.

## Results

### Clinical features

The demographics information for cases and controls were displayed in Table 2. The healthy cohort comprised 456 males and 462 females, with a mean age of 39.73 ± 10.06 years. While the AAU patient group included 211 males and 209 females, with a mean age of 40.94 ± 11.86 years. Among the 353 AAU patients who underwent HLA-B27 assessment, a total of 241 individuals tested positive (Table 2). The statistical analysis revealed no appreciable difference in age or gender was found between the cases and controls (*P* > 0.05).

**Table 2:** Clinical characteristics, age and gender distribution in AAU subjects and healthy individuals

Clinical Features	Total	%
AAU patients	420	
Age at onset, year ± SD	40.94 ± 11.86	
Male	211	50.2
Female	209	49.8
HLA-B27 (+)	241	57.4
HLA-B27 (-)	112	26.7
Controls	918	
Age at onset, year ± SD	39.73 ± 10.06	
Male	456	49.7
Female	462	50.3

SD = standard deviation;

AAU = Acute anterior uveitis

### Genotype results

Ten SNPs covering *IL10* and *IL6R* genes were successfully genotyped, with each SNP exhibiting a genotyping success rate exceeding 95%. None of them deviated from the HWE in controls (*P* > 0.05). P_uncorr_ values showed weak associations between AAU and four SNPs. In *IL6R*/rs4845618, the frequency of TT genotype (*P* = 0.034, OR = 1.329, 95%CI = 1.021–1.729) was increased in AAU subjects versus healthy participants while the frequency of GT phenotype (*P* = 0.028, OR = 0.772, 95%CI = 0.612–0.973) was decreased (Table 3). In the *IL10* gene, a preliminary association between rs1800871 and AAU was also observed with the allele frequency of G being lower (*P* = 0.035, OR = 0.824, 95%CI = 0.688–0.987) in AAU subjects as compared with controls. Additionally, the GG genotype in *IL10*/rs3021094 and the AA genotype in *IL10*/rs2222202 showed a minor increased trends in AAU patients (*P* = 0.011, OR = 1.417, 95%CI = 1.083–1.853; *P* = 0.012, OR = 1.204, 95%CI = 0.955–1.518, respectively). However, after the Bonferroni corrections were performed, all of the positive results that were mentioned earlier disappeared (Table 4). The remaining 6 SNPs had no significant correlation with AAU (see supplemental Table 1).

**Table 3:** Polymorphisms of *IL6R/rs4845618* in AAU.

	Genotype	Cases, No.		Controls, No.					
SNPs	Allele	(Frequency)		(Frequency)		*P* Value	Pc Value	OR (95% CI)	
**rs4845618** ***(IL-6R)***	GG	106	(0.252)	220	(0.241)	6.68E-01	*NS*	1.060 (0.812 to 1.385)	
	GT	196	(0.467)	484	(0.531)	2.84E-02	*NS*	0.772 (0.612 to 0.973)	
	TT	118	(0.281)	207	(0.227)	3.40E-02	*NS*	1.329 (1.021 to 1.729)	
	G	408	(0.486)	924	(0.507)	3.04E-01	*NS*	0.918 (0.779 to 1.081)	
	T	432	(0.514)	898	(0.493)	3.04E-01	*NS*	1.089 (0.925 to 1.283)	

NS = no significant difference; OR = odds ratio; *P*_c_ = *P* value with Bonferroni correction;

SNP = single nucleotide polymorphism

**Table 4:** Polymorphisms of *IL10/rs1800871, rs3021094 and rs2222202* in AAU.

	Genotype	Cases, No.		Controls, No.					
SNPs	Allele	(Frequency)		(Frequency)		*P* Value	Pc Value	OR (95% CI)	
**rs1800871** ***(IL-10)***	GG	28	(0.067)	90	(0.099)	5.61E-02	*NS*	0.652 (0.420 to 1.014)	
	GA	178	(0.425)	402	(0.442)	5.63E-02	*NS*	0.933 (0.739 to 1.179)	
	AA	213	(0.508)	418	(0.459)	9.64E-02	*NS*	1.217 (0.965 to 1.534)	
	G	234	(0.279)	582	(0.320)	3.52E-02	*NS*	0.824 (0.688 to 0.987)	
	A	604	(0.721)	1238	(0.680)	3.52E-02	*NS*	1.213 (1.013 to 1.453)	
**rs3021094** ***(IL-10)***	GG	114	(0.273)	189	(0.209)	1.08E-02	*NS*	1.417 (1.083 to 1.853)	
	GT	202	(0.483)	475	(0.526)	1.48E-01	*NS*	0.843 (0.668 to 1.063)	
	TT	102	(0.244)	239	(0.265)	4.25E-01	*NS*	0.897 (0.686 to 1.172)	
	G	430	(0.514)	853	(0.472)	4.43E-02	*NS*	1.183 (1.004 to 1.394)	
	T	406	(0.486)	953	(0.528)	4.43E-02	*NS*	0.845 (0.717 to 0.996)	
**rs2222202** ***(IL-10)***	CC	28	(0.067)	88	(0.097)	6.53E-02	*NS*	0.662 (0.425 to 1.029)	
	CA	180	(0.429)	401	(0.444)	5.97E-01	*NS*	0.939 (0.743 to 1.186)	
	AA	212	(0.505)	414	(0.458)	1.16E-01	*NS*	1.204 (0.955 to 1.518)	
	C	236	(0.281)	577	(0.319)	4.55E-02	*NS*	0.832 (0.695 to 0.996)	
	A	604	(0.719)	1229	(0.681)	4.55E-02	*NS*	1.202 (1.004 to 1.439)	

NS = no significant difference; OR = odds ratio; *P*_c_ = *P* value with Bonferroni correction;

SNP = single nucleotide polymorphism

Given the significant association between AAU and HLA-B27, we have performed a further stratified analysis on the genotyping results of ten loci based on the AAU patients’ HLA-B27 outcomes. Unfortunately, after the Bonferroni corrections, we did not find any evidence that the ten SNPs of *IL10*/(rs1800871, rs3790622, rs1800896, rs3021094 and rs2222202) and *IL6R*/( rs2228145, rs6690230, rs4845618, rs4845374 and rs4129267) were associated with the HLA-B27^+^ AAU risk (Pc > 0.05) (see supplemental Table 2).

### The haplotype analysis

The association between haplotypes of the ten SNPs and AAU was investigated by means of analysis. After applying the correction, there was no association observed. The comprehensive information regarding haplotype assessment was displayed in Table 5.

**Table 5:** The haplotype analysis of the ten SNPs.

	Haplotype	Frequency (%)		P Value	OR (95% CI)
		Case	Control		
**AAU vs. Controls**	CATCAATGGA	37.79 (0.047)	59.87 (0.036)	0.286	1.255 (0.827 to 1.905)
	CTGCAATGGA	31.33 (0.039)	88.99 (0.054)	0.067	0.678 (0.447 to 1.030)
	CTGCAATGTA	27.18 (0.034)	33.75 (0.020)	0.068	1.606 (0.962 to 2.684)
	GTGCAATAGA	31.17 (0.039)	50.99 (0.031)	0.408	1.212 (0.769 to 1.909)
	GTGCAATGGA	124.61 (0.155)	234.42 (0.142)	0.661	1.055 (0.831 to 1.339)
	GTGCAATGTA	74.28 (0.092)	136.21 (0.083)	0.609	1.081 (0.802 to 1.456)
	GTGCAGCGTC	15.75 (0.020)	51.23 (0.031)	0.073	0.596 (0.336 to 1.056)
	GTGCAGTGTC	62.68 (0.078)	146.80 (0.089)	0.222	0.825 (0.605 to 1.124)
	GTTTCATGGA	129.57 (0.161)	243.43 (0.148)	0.644	1.057 (0.836 to 1.337)
	GTTTCATGTA	59.53 (0.074)	106.28 (0.064)	0.533	1.111 (0.798 to 1.546)
	GTTTCGCGTC	27.75 (0.035)	40.64 (0.025)	0.222	1.357 (0.830 to 2.217)
	GTTTCGTGTC	76.52 (0.095)	178.87 (0.109)	0.177	0.823 (0.619 to 1.093)

The frequency < 0.03 was ignored in analysis

OR = odds ratio

## Discussion

In our study, genotyping analysis of 420 AAU subjects and 918 healthy individuals were conducted for ten SNPs in *IL10* and *IL6R* genes, in a Chinese Han population. Although four SNPs, *IL6R*/rs4845618, *IL10*/rs1800871, *IL10*/rs3021094 and *IL10*/rs2222202 showed preliminary associations with AAU, the significance was no longer present after applying the Bonferroni correction. Furthermore, analysis was carried out to study the correlation of LD block of the ten SNPs with AAU, and no association was observed.

AAU is considered as an inflammatory ocular disease, and its recurrent attacks can cause severe visual impairment or even blindness. While the precise cause of the condition remains unknown, extensive research suggests that immune and genetic components may both contribute to its development [22, 23]. Although HLA-B27 has been linked to AAU strongly, not all HLA-B27 positive subjects will develop the disease, suggesting the involvement of additional genes [23, 24]. Recent association studies have uncovered numerous susceptibility genes outside of the MHC region, such as the *IL23R*, *FOXO1*, *TRAF5* and *TNFSF15* genes [6–8, 21]. However, further investigation is necessary to gain a greater understanding of the genetic foundation of the disease.

IL10 is a vital immunoregulatory cytokine for inflammatory and immune responses [11]. Low IL10 expression has been linked to the etiology of inflammatory and autoimmune illnesses [12] including Behcet’s disease (BD), and polymorphisms in multiple immunoregulatory genes have been linked to an increased risk of developing BD [25]. IL6R is a key member of IL-6 signal pathway and has been linked to various autoimmune and inflammatory conditions (including IBM, AS and RA). A humanized antibody against the IL6 receptor called tocilizumab has displayed remarkable clinical effectiveness in the treatment of RA and JIA [10, 25]. *IL6R* gene may be involved in the development and treatment of autoimmune diseases including uveitis [10, 26]. Taking into account of aforementioned discoveries, we have developed a significant interest in the genetic roles of *IL6R* and *IL10* in AAU.

As is well-known, selecting candidate SNPs is critical for gene variation study. In our research, ten SNPs covering *IL10* and *IL6R* genes were chosen based on previous association studies of autoimmune disorders, such as IBD, RA, MS and AS. *IL10*/rs1800896, rs1800871, rs2222202 and rs3790622 have been found to be associated with CD in several studies [11, 12]. Moreover, rs3021094 and rs1800871 have both been demonstrated to be associated with BD in the Chinese population [13]. According to a GWAS investigation within the Caucasian demographic, *IL6R*/rs6690230 C allele has been identified to be a protective element against AS and AAU [21]. Multiple analyses have demonstrated a notable link between *IL6R*/rs4845618 and *IL6R*/rs4845374 and RA [27]. Various research has established a connection between *IL6R*/rs2228145 and an array of autoimmune conditions, including IBD and RA [19]. A study conducted on genetic polymorphisms revealed a significant correlation between *IL6R*/rs4129267 and AS [20]. In our study, *IL10*/rs1800871, rs3021094, rs2222202 and *IL6R*/rs4845618 showed a weak association with AAU, but unfortunately, this association disappeared after correction.

To ensure robustness of data, a number of efforts have been made. Firstly, the diagnosis of AAU patients was strictly based on their symptoms (such as ocular pain, red eyes, and vision loss), clinical examinations (such as keratic precipitates, ciliary congestion, and anterior chamber cells) as well as instrumental tests (ultrasound biomicroscopy and anterior segment image). Patients with any uncertainty in their diagnoses were not included in this study. Secondly, all patients were all in the acute phase of the disorder and none of the patients had a history of any other autoimmune disorders. Thirdly, all AAU patients come from a Chinese Han population in order to prevent racial bias in the outcomes. Age and gender were matched between the patients and controls.

All the above-mentioned efforts have made the results reliable. According to the current investigation, none of the tested ten SNPs of *IL10* and *IL6R* genes were connected to AAU. However, it is worthwhile to point out that our study does not rule out the possibility of an association between these SNPs and AAU in other ethnic. Additionally, it is unknown whether there is an association between other SNPs on *IL10* and *IL6R* genes and AAU, which should be investigated in future research.

## Conclusions

Our findings suggested that polymorphisms of the tested ten SNPs of *IL10* and *IL6R* genes did not have any correlation with the susceptibility to AAU among the Han Chinese population.

## Data Availability

The authors confirm that the data supporting the findings of this study are available within the article (tables and supplementary table), Further request about the data of this study are available from the corresponding author on reasonable request (dulplab@live.cn).

## Abbreviations

AAU: Acute anterior uveitis
IL6R: Interleukin-6 receptor
IL10: Interleukin-10
MS: Multiple sclerosis
IBD: Inflammatory bowel disease
T1D: Type 1 diabetes
*RA*: Rheumatoid arthritis
*BD*: Behcet’s disease
AS: Ankylosing spondylitis
SS: Systemic sclerosis
LD: Linkage disequilibrium
